# The Impact of *Bartonella* VirB/VirD4 Type IV Secretion System Effectors on Eukaryotic Host Cells

**DOI:** 10.3389/fmicb.2021.762582

**Published:** 2021-12-15

**Authors:** Katja Fromm, Christoph Dehio

**Affiliations:** Biozentrum, University of Basel, Basel, Switzerland

**Keywords:** host-pathogen interaction, bacterial pathogenesis, type IV secretion system (T4SS), VirB/VirD4, bacterial effector protein, *Bartonella* effector protein (Bep)

## Abstract

*Bartonella* spp. are facultative intracellular pathogens that infect a wide range of mammalian hosts including humans. The VirB/VirD4 type IV secretion system (T4SS) is a key virulence factor utilized to translocate *Bartonella* effector proteins (Beps) into host cells in order to subvert their functions. Crucial for effector translocation is the C-terminal Bep intracellular delivery (BID) domain that together with a positively charged tail sequence forms a bipartite translocation signal. Multiple BID domains also evolved secondary effector functions within host cells. The majority of Beps possess an N-terminal filamentation induced by cAMP (FIC) domain and a central connecting oligonucleotide binding (OB) fold. FIC domains typically mediate AMPylation or related post-translational modifications of target proteins. Some Beps harbor other functional modules, such as tandem-repeated tyrosine-phosphorylation (EPIYA-related) motifs. Within host cells the EPIYA-related motifs are phosphorylated, which facilitates the interaction with host signaling proteins. In this review, we will summarize our current knowledge on the molecular functions of the different domains present in Beps and highlight examples of Bep-dependent host cell modulation.

## Introduction

Bacterial type IV secretion systems (T4SSs) are multi-protein complexes embedded in the cell envelopes of bacteria and some archaea. These systems enable the translocation of macromolecules across membranes, such as uptake of extracellular DNA and translocation of protein effectors into recipient cells (Christie et al., 2014; Waksman, 2019; Costa et al., 2020). T4SSs are essential for the pathogenicity of many bacteria infecting humans and other mammals, such as *Helicobacter pylori*, *Legionella pneumophila*, *Brucella* spp. or *Bartonella* spp. (Cascales and Christie, 2003). Based on structural characteristics T4SS can be categorized into T4AS and T4BS systems. T4AS are composed of 12 subunits termed VirB1-11 and VirD4 according to the nomenclature of the paradigmatic VirB/VirD4 T4SS of the plant pathogen *Agrobacterium tumefaciens* (Waksman, 2019). VirB2-11 assemble the translocation channel spanning through the inner and outer membranes. Typically, the membrane-bound ATPase VirD4, also termed type IV secretion (T4S) coupling protein (T4CP), recognizes T4S substrates prior to translocation. The majority of the T4S substrates contain signals at their C-termini consisting of a few positively charged or hydrophobic residues (Christie et al., 2014). However, some T4S substrates form a larger structural scaffold as translocation signal. These include the R1-plasmid encoded relaxase TraI (Redzej et al., 2013) and the effector proteins of *Bartonella* spp. (Schulein et al., 2005).

**FIGURE 1:**
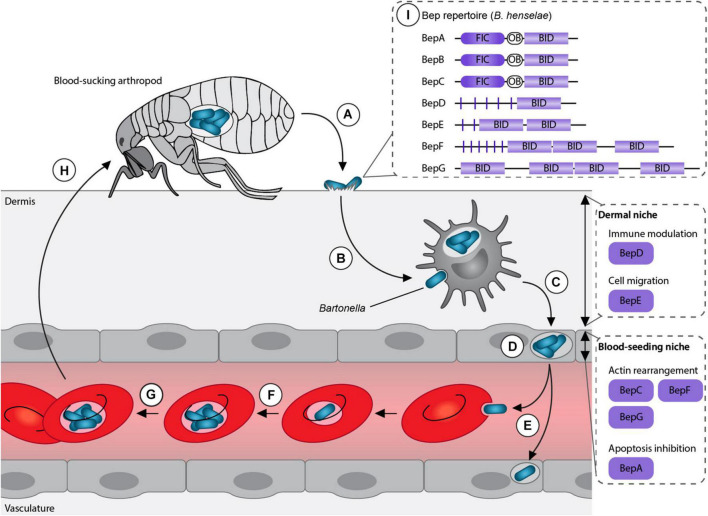
Model of *Bartonella* life cycle. **(A)** Bartonellae replicate inside the midgut of their arthropod vector and are secreted with their feces. **(B)** After inoculation into the dermis, the bacteria colonize the “dermal niche,” which most likely includes dendritic cells. During this infection stage, the downregulation of the immune response mediated by BepD might play an essential role. **(C)** Migratory immune cells are considered to disseminate the bacteria to the “blood-seeding niche,” a process that seems to depend on BepE. **(D)** Inside the “blood-seeding niche,” the bacteria likely colonize endothelial cells, which might require the action of BepC, BepF, BepG, and BepA. **(E)** From the “blood-seeding niche” Bartonellae are disseminated into the blood stream, where they invade erythrocytes. **(F)** The bacteria undergo replication and **(G)** persist until **(H)** they can be taken up during the next blood meal by another arthropod. **(I)** Representative *Bartonella* effector protein (Bep) repertoire of the model organism *B. henselae*. Adapted from Siamer and Dehio (2015).

Bartonellae are Gram-negative facultative intracellular pathogens, which infect various mammals including humans. These bacteria are transmitted by blood-sucking arthropods such as fleas, sand flies or lice. The current model suggests that the bacteria are superficially inoculated into the dermis (e.g., through scratching) followed by the colonization of two sequential niches, the “dermal niche” and the “blood-seeding niche” (Okujava et al., 2014; Siamer and Dehio, 2015; Figure 1). In the dermal stage of infection, the bacteria might hijack migratory cells, such as dendritic cells or macrophages, to reach the “blood-seeding niche.” In this niche, the bacteria colonize endothelial cells and possibly other cell types. Subsequently, *Bartonella* seed into the blood stream where they cause a long-lasting intraerythrocytic bacteremia, an infection stage restricted to the natural reservoir host (Schulein et al., 2001; Harms and Dehio, 2012; Pulliainen and Dehio, 2012).

The genus *Bartonella* can be divided into three phylogenetic clades: *Bartonella apis* and *Bartonella tamiae*, which occupy ancestral positions and the Eubartonellae, which are further divided into four distinct lineages (Engel et al., 2011; Guy et al., 2013). *Bartonella anchashensis* of lineage 1 and all members of lineages 3 and 4 harbor a VirB/VirD4 T4SS (Harms et al., 2017). The VirB/VirD4 T4SS in *Bartonella* is essential for successful host colonization. T4SS-deficient mutants of *Bartonella tribocorum*, Δ*virB4* or Δ*virD4*, failed to invade the blood stream in an experimental rat infection model (Schulein and Dehio, 2002). Multiple *in vitro* studies with the major human pathogen *Bartonella henselae* showed that *Bartonella* effector proteins (Beps) are translocated *via* the VirB/VirD4 T4SS into different host cells belonging to the “dermal” and the “blood-seeding niche.” Inside the host cells, Beps target various components to modulate the immune response and to subvert host cellular functions to the benefit of the pathogen (Schulein et al., 2005; Schmid M. C. et al., 2006; Okujava et al., 2014; Sorg et al., 2020; Marlaire and Dehio, 2021; Figure 1).

In this review, we will discuss recent advances made concerning the functional role of Beps during host colonization. We will focus on the structural and functional aspects of different domains present in Beps with regard to the subversion of host cellular function.

## Domain Architecture of *Bartonella* Effector Proteins

*Bartonella* effector proteins display a modular domain architecture (Figure 1). The majority of the Beps possess an N-terminal filamentation induced by cAMP (FIC) domain followed by a central connecting oligonucleotide binding (OB) fold and a C-terminal Bep intracellular delivery (BID) domain (Engel et al., 2011; Harms et al., 2017). Instead of the FIC domain, some effectors harbor tandem-repeated tyrosine phosphorylation motifs (pY) and/or additional BID domains (Wagner and Dehio, 2019; Figure 1). While the C-terminal BID domains function as a conserved T4S signal, some BID domains also acquired secondary effector functions within eukaryotic host cells (Truttmann et al., 2011a; Pulliainen et al., 2012; Okujava et al., 2014).

The different domain architectures suggest that Beps evolved from a single ancestral effector with a FIC-OB-BID structure *via* independent gene duplication and recombination events. Diverse Bep repertoires arose in the three distinct lineages resulting in Bep197-234 in *Bartonella ancashensis* of lineage 1, Bep1-10 in bacteria of lineage 3, and BepA-I in lineage 4 (Harms et al., 2017).

## Structural Features of BID Domains

*Bartonella* effector proteins are recognized by the VirB/VirD4 T4SS *via* a bipartite translocation signal composed of the approximately 100-aa-long BID domain and a short positively charged C-terminal tail (Schulein et al., 2005; Stanger et al., 2017). BID domains are sequence-variable but adopt a conserved structural fold consisting of an anti-parallel four-helix bundle topped with a hook (Stanger et al., 2017; Wagner et al., 2019). The conserved fold and the elongated shape of the BID domains might be crucial features for the secretion signal. In addition, the surface charge distribution of BID domains is also to some degree conserved, displaying two positively charged areas separated by one negatively charged patch (Stanger et al., 2017; Wagner et al., 2019). The conserved fold of the BID domains might be essential for the secretion *via* the VirB/VirD4 T4SS, while the highly variable sequence might enable the acquisition of secondary functions.

### BID Domain-Mediated Host Cell Modulations

The secondary evolved functions of some BID domains are essential for host colonization at various stages of the infection cycle. These functions include dissemination within the host, bacterial uptake through induction of stress fiber formation and inhibition of apoptosis.

**FIGURE 2:**
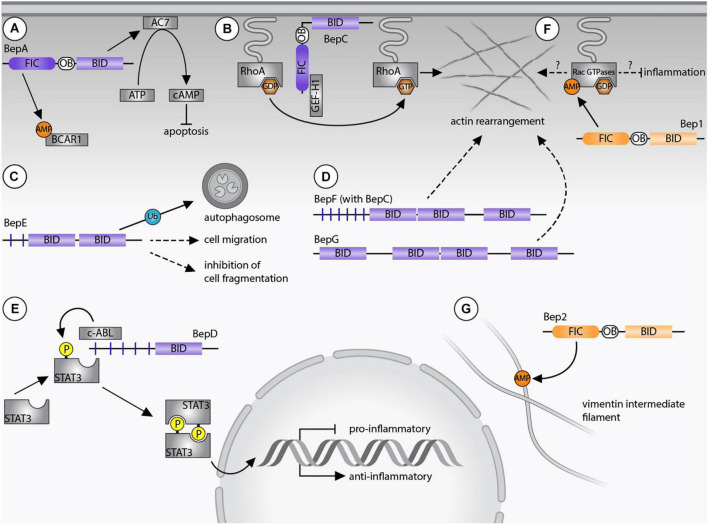
*Bartonella* effector protein-mediated subversion of host cellular functions. **(A)** BepA AMPylates breast cancer anti-estrogen resistance protein 1 (BCAR1) *via* its filamentation induced by cAMP (FIC) domain and interacts with adenylyl cyclase 7 (AC7) *via* its Bep intracellular delivery (BID) domain. The AC7-mediated conversion of ATP to cAMP results in inhibition of apoptosis. **(B)** BepC recruits GEF-H1 *via* its FIC domain to activate the RhoA pathway. **(C)** BepE is required for the dissemination within the mammalian host and promotes cell migration. The BID domain of BepE of *B. quintana* becomes ubiquitinated inside host cells and is degraded by autophagy. **(D)** BepG or BepF together with BepC induce stress fiber formation resulting in the formation of the invasome. **(E)** Upregulation of an anti-inflammatory immune response depends on the pY-domain of BepD, which provides a binding platform for the SH2 domain containing proteins c-ABL and STAT3. Due to close proximity, STAT3 becomes phosphorylated by c-ABL and triggers gene expression of IL-10. **(F)** Bep1 AMPylates the GDP-bound Rac-subfamily of GTPases. **(G)** Bep2 AMPylates the vimentin intermediate filaments. Dashed arrows indicate currently unknown target proteins, continuous arrows display interactions with confirmed targets. *Bartonella* effector proteins (Beps) of lineage 4 of are displayed in purple, Beps of lineage 3 Bartonellae are shown in orange.

*Bartonella* spp. supposedly infect dendritic cells during the dermal stage of infection in order to reach the “blood-seeding niche.” BepE was shown to promote the migratory capability of dendritic cells indicating that the bacteria exploit these cells as Trojan horses in order to reach the blood stream. Further, dissemination of *B. tribocorum* into the blood stream depends on the function of BepE (Okujava et al., 2014). BepE of *B. henselae* harbors a pY-domain and two BID domains (Wagner et al., 2019). Although, the pY motif of BepE interacts with several host proteins (Selbach et al., 2009), the dissemination of the bacteria into the blood stream exclusively relies on the BID domains. *In vitro* assays demonstrated that the terminal BID domain of BepE was required to safeguard dendritic cells from damage triggered by BepC (Okujava et al., 2014; Figure 2).

A recent publication demonstrated that the BID domains of BepE (*Bartonella quintana*) are ubiquitinated and trigger selective autophagy inside the host cells. Interestingly, BepE of *B. henselae* was not ubiquitinated (Wang et al., 2019). These data indicate that orthologous Beps can vary between *Bartonella* species. Future studies should aim to clarify whether the assigned functional differences are relevant in a pathogen- or host-specific context.

*B. henselae* invades endothelial cells either individually by endocytosis or as bacterial aggregate through the formation of the so-called invasome. This unique cellular structure is induced by F-actin rearrangements and stress fiber formation (Dehio, 1999). The invasome formation depends on the VirB/VirD4 T4SS and is induced by either BepG or the combined action of BepC and BepF (Schmid et al., 2004; Rhomberg et al., 2009; Truttmann et al., 2011b). BepF of *B. henselae* contains three BID domains. The two non-terminal BID domains trigger the invasome formation (together with BepC), while the third BID domain is negligible in this process (Truttmann et al., 2011a; Figure 2). BepG consists of solely four BID domains connected *via* short linker sequences, hence it is likely that at least one of them induces the invasome formation (Rhomberg et al., 2009).

*B. henselae* and *B. quintana* promote the proliferation of human endothelial cells by inhibiting apoptosis (Kirby and Nekorchuk, 2002; Schmid M. C. et al., 2006; Pulliainen et al., 2012). The anti-apoptotic activity depends on the BID domain of BepA, which interacts with the catalytic subunit C2 of the human adenylyl cyclase 7 (AC7) (Pulliainen et al., 2012). AC7 is a plasma membrane-bound protein that regulates cAMP synthesis (Sadana and Dessauer, 2009). The interaction of BepA with C2 likely enhances the association with the C1 subunit and thereby potentiates cAMP production. The elevated cAMP-levels and consecutive upregulation of gene expression then leads to the inhibition of apoptosis (Pulliainen et al., 2012; Figure 2). It is believed that the bacteria undergo several rounds of replication within “primary” or “blood-seeding niche” before invading the blood stream (Schulein et al., 2001). Inhibition of endothelial cell death might therefore be crucial for host colonization. Interestingly, the BepA ortholog of *B. tribocorum* did not display anti-apoptotic activity (Schmid D. et al., 2006), indicating again that orthologous Beps can fulfill divergent functions in the infection process.

## Evolutionary Diversification of FIC Domain-Containing *Bartonella* Effector Proteins

FIC domains are characterized by a helical topology and a conserved FIC signature motif. The canonical signature motif [HPFx(D/E)GNGRxxR] comprises a catalytic histidine and residues involved in adenosine triphosphate (ATP) binding. This motif is strictly required for catalyzing the AMPylation reaction, a post-translational modification that involves the transfer of an adenosine monophosphate (AMP) moiety from ATP onto target proteins (Engel et al., 2012; Roy and Cherfils, 2015; Harms et al., 2016). While most FIC domain-containing proteins harbor a canonical FIC signature motif, several others also carry non-canonical FIC signature motifs, which may mediate different post-translational modifications (Mukherjee et al., 2011; Castro-Roa et al., 2013; Roy and Cherfils, 2015). The deAMPylating activity of some FIC enzymes was also demonstrated (Perera et al., 2019; Veyron et al., 2019). Additionally, FIC domains contain a β-hairpin, commonly also referred to as “flap,” which is involved in substrate binding (Xiao et al., 2010). Comparison of crystal structures of FIC domains of various Beps revealed a highly conserved conformation. However, the flap region is less conserved, which may relate to the individual target spectra of different FIC enzymes. Based on the canonical FIC signature motif, it was speculated that around half of the investigated Beps might represent AMP transferases, such as Bep1, Bep2 and BepA (Schirmer et al., 2021).

### AMPylating FIC Domain-Containing *Bartonella* Effector Proteins

FIC domains containing a canonical FIC signature motif are likely AMPylators, but their targets are diverse. BepA of *B. henselae* AMPylates the breast cancer anti-estrogen resistance protein 1 (BCAR1) (Gulen et al., 2020) and additional unidentified proteins (Palanivelu et al., 2011).

Bep2 AMPylates the intermediate filament protein vimentin (Pieles et al., 2014), while the closely related Bep1 AMPylates Rho family GTPases. Similarly, FIC domain-containing effectors of other pathogens are known to AMPylate Rho GTPases to regulate the cytoskeleton or the immune response of mammalian host cells. Prominent examples are IbpA of *Histophilus somni* and VopS of *Vibrio parahaemolyticus* (Yarbrough et al., 2009; Mattoo et al., 2011). AMPylation of those GTPases blocks downstream signaling cascades and ultimately leads to a collapse of the cytoskeleton and cell death (Roy and Cherfils, 2015). What distinguishes Bep1 of *Bartonella rochalimae* from these FIC enzymes with broad target spectrum is the selectivity for the Rac-subfamily of Rho GTPases (i.e., Rac1/2/3 and RhoG). As such, Bep1 is the first bacterial effector selectively targeting Rac-subfamily GTPases without affecting the Rho GTPase Cdc42. Bep1 modifies Y32 of Rac1, a residue involved in GTP binding, limiting its physiological targets to GDP-bound GTPases (Dietz et al., 2021; Figure 2).

The target selectivity of Bep1 is based on a short insert of six residues in the flap loop of the FIC domain. A modeled complex between the FIC domain of Bep1 and Rac2 revealed that the extended flap of Bep1 interacts with the nucleotide-binding G4 motif [(K/Q)xD] and the following Rho-insert helix of Rac2. Crucial for the target selectivity of Bep1 are two identified salt bridges between a conserved lysine residue in the G4 motif and a glutamate in the Rho-insert. These residues are only present in the Rac-subfamily GTPases (Dietz et al., 2021). The Bep1 target selectivity might play a critical role for the evasion of the innate immune response. In fact, inhibition of Rac1 and Rac2 decreases the production of reactive oxygen species (ROS), which interferes with clearance of bacterial infections (Kurkchubasche et al., 2001; Bhattacharyya et al., 2014). In contrast, inhibition of RhoA, as for example by IbpA or VopS, triggers the activation of the pyrin inflammasome resulting in an inflammatory type of programmed cell death called pyroptosis (Xu et al., 2014; Heilig and Broz, 2018). Thus, inhibition of Rac GTPases but avoiding RhoA inhibition may be crucial for *Bartonella* to evade the innate immune response and to establish chronic infections. However, experiments showing the Bep1-specific inhibition of Rac-subfamily GTPases inside host cells are still missing.

### Functions of Non-canonical FIC Domains

BepC contains a non-canonical FIC signature motif, which differs from the canonical motif by the replacement of an acetic residue (D/E) by a lysine. This FIC domain should thus be devoid of AMPylation activity, but might encode a different enzymatic activity. Recently, the molecular mechanism underlying BepC-dependent stress fiber formation has been uncovered. After translocation into host cells, BepC localizes to the plasma membrane *via* its BID domain. Immunoprecipitation revealed that BepC interacts with GEF-H1 *via* its FIC domain. GEF-H1 is a guanine nucleotide exchange (GEF) factor that remains inactive when bound to microtubules. Upon release, GEF-H1 activates the RhoA pathway. The interaction of BepC and GEF-H1 might not depend on post-translational modifications. Extensive mutagenesis of the conserved non-canonical FIC motif of BepC did not interfere with stress fiber formation. Rather, the BepC-triggered relocalization of GEF-H1 to the plasma membrane leads to activation of RhoA by exchanging GDP to GTP (Figure 2). In turn, the downstream Rho kinase ROCK is activated and induces stress fiber formation (Marlaire and Dehio, 2021; Wang et al., 2021).

## pY Domain-Dependent Modulation of the Innate Immune Response

In order to colonize their hosts pathogens evolved various mechanisms to modulate the innate immune response (Reddick and Alto, 2014). The transcription factor Signal Transducer and Activator of Transcription 3 (STAT3), a central regulator of inflammation, mediates the switch from a pro- to an anti-inflammatory immune response (Hillmer et al., 2016; Huynh et al., 2019). In response to cytokine signaling, Janus kinases (JAKs) phosphorylate STAT3 on Y705, causing its dimerization and translocation into the nucleus, where it activates gene transcription (Huynh et al., 2019). Alternatively, STAT3 becomes phosphorylated by the Abelson tyrosine kinase (c-ABL) (Allen et al., 2011). The canonical JAK-STAT3 signaling pathway controls the expression of pro-inflammatory cytokines (e.g., IL-6, TNF-α) and anti-inflammatory cytokines (like IL-10) (Williams et al., 2004; Melillo et al., 2010). Recent evidence suggests that pathogens evolved strategies to modulate innate immunity by STAT3 activation (Gibbs et al., 2020; Panagi et al., 2020).

BepD promotes an anti-inflammatory response through an exceptional pathway for STAT3 activation. BepD of *B. henselae* harbors two almost identical tyrosine phosphorylation domains (pY and pY’), each with nine EPIYA-related motifs which were originally identified in CagA of *H. pylori* (Backert and Selbach, 2005; Hayashi et al., 2013). Inside host cells, tyrosine phosphorylation of the respective EPIYA-related motifs enables interaction with SH2-domain containing proteins (Selbach et al., 2009; Sorg et al., 2020). c-ABL and STAT3 were identified as interaction partners of BepD. Moreover, STAT3 was found to be phosphorylated on Y705. Interestingly, the BepD-dependent activation of STAT3 occurred independently of autocrine or paracrine cytokine signaling. Accordingly, JAKs, which integrate pro- and anti-inflammatory cytokine signaling to phosphorylate STAT3, were not required for BepD-dependent STAT3 activation. Instead, c-ABL recruited to the phosphorylated EPIYA motifs of BepD triggered the phosphorylation of STAT3 on Y705. Through the activation of STAT3, BepD impairs the pro-inflammatory response and promotes secretion of the anti-inflammatory cytokine IL-10 (Sorg et al., 2020; Figure 2). This mechanism might be important for *Bartonella* to modulate innate immune cells encountered in the dermal niche.

## Concluding Remarks and Open Questions

The stealth infection strategy of *Bartonella* requires precise modulation of the host cellular function in order to invade the blood stream. Throughout the different stages of infection, the translocation of Beps *via* the VirB/VirD4 T4SS into various host cells favors the pathogenicity of the bacteria (Figure 1). BepD and BepE seem to support the progress from the dermal site of infection towards the “blood-seeding niche” by downregulating the innate immune response and safeguarding the migratory capacity of hijacked cells against deleterious effects by BepC. Colonization of endothelial cells is enhanced by the combined action of BepG and BepC together with BepF, which induce internalization of bacterial aggregates. BepA inhibits apoptosis of endothelial cells, which constitute the “blood-seeding niche.”

Most information concerning the function of Beps was gathered using the cat-adapted strain *B. henselae* that incidentally infects humans but not rodents. However, an experimental model to study the infection of cats is rather laborious (Chomel et al., 1996; Foil et al., 1998). Therefore, *in vivo* studies in the natural host were often conducted with rodent-specific strains (Boulouis et al., 2001; Schulein and Dehio, 2002). However, *in vitro* infection protocols to study the effector function of rodent-specific strains are missing. *Bartonella* species translocate individual cocktails of Beps into the host cells possibly affecting different host cellular processes (Harms et al., 2017). While different orthologs of BepD share a conserved function (Sorg et al., 2020), other Beps seem to differ in a species-specific context, as for example BepA and BepE (Schmid D. et al., 2006; Wang et al., 2019). Suitable *in vitro* and *in vivo* models using the same *Bartonella* species will be necessary to study the role of Beps in the natural host.

Despite the progress made to elucidate the function of lineage 4 Beps, less is known about Beps from lineage 3. Bep1 and Bep2 AMPylate several host proteins that potentially affect the cytoskeleton (Figure 2). However, *in vitro* or *in vivo* assays demonstrating their function inside host cells or a suitable model organism to study *Bartonella* species of lineage 3 are still missing.

Next to the need to solve the functions of several uncharacterized Beps, the translocation mechanism through the VirB/VirD4 T4SS should be also investigated in more detail. The BID domain and the positively charged tail are essential for the secretion of Beps *via* the T4SS (Schulein et al., 2005; Schmid M. C. et al., 2006), however, structural information resolving the interaction of Beps with the T4CP is still missing. Future work should aim to determine key residues of BID domains mediating the interaction with VirD4.

## Author Contributions

KF designed the figures. KF and CD wrote the manuscript. Both authors contributed to the article and approved the submitted version.

## Conflict of Interest

The authors declare that the research was conducted in the absence of any commercial or financial relationships that could be construed as a potential conflict of interest.

## Publisher’s Note

All claims expressed in this article are solely those of the authors and do not necessarily represent those of their affiliated organizations, or those of the publisher, the editors and the reviewers. Any product that may be evaluated in this article, or claim that may be made by its manufacturer, is not guaranteed or endorsed by the publisher.
